# Quantitative assessment of sitting time in ambulant adults with Muscular Dystrophy

**DOI:** 10.1371/journal.pone.0260491

**Published:** 2021-11-19

**Authors:** Matthew F. Jacques, Gladys L. Onambele-Pearson, Bryn Edwards, Christian G. De Goede, Christopher I. Morse

**Affiliations:** 1 Faculty of Science and Engineering, Musculoskeletal Science & Sports Medicine Research Centre, School of Healthcare Science, Manchester Metropolitan University, Manchester, United Kingdom; 2 School of Life Sciences, Queens Medical Centre, Nottingham, United Kingdom; 3 The Neuromuscular Centre, Winsford, Cheshire, United Kingdom; 4 Royal Preston Hospital, Preston, Lancashire, United Kingdom; UCL: University College London, UNITED KINGDOM

## Abstract

**Background:**

Current investigations into physical behaviour in Muscular Dystrophy (MD) have focussed largely on physical activity (PA). Negative health behaviours such as sedentary behaviour (Physical Behaviour) and sitting time (Posture Classification) are widely recognised to negatively influence health, but by contrast are poorly reported, yet could be easier behaviours to modify.

**Methods:**

14 ambulant men with MD and 12 healthy controls (CTRL) subjects completed 7-days of free-living with wrist-worn accelerometry, assessing physical behaviour (SB or PA) and Posture Classification (Sitting or Standing), presented at absolute (minutes) or relative (% Waking Hours). Participant body composition (Fat Mass and Fat Free Mass) were assessed by Bioelectrical Impedance, while functional status was assessed by 10 m walk test and a functional scale (Swinyard Scale).

**Results:**

Absolute Sedentary Behaviour (2.2 Hours, *p* = 0.025) and Sitting Time (1.9 Hours, *p* = 0.030 was greater in adults with MD compared to CTRL and Absolute Physical Activity (3.4 Hours, *p* < 0.001) and Standing Time (3.2 Hours, *p* < 0.001) was lower in adults with MD compared to CTRL. Absolute hours of SB was associated with Fat Mass (Kg) (R = 0.643, *p* < 0.05) in ambulatory adults with MD,

**Discussion:**

This study has demonstrated increased Sedentary Behaviour (2.2 hours) and Sitting time (1.9 Hours) in adults with MD compared to healthy controls. Extended waking hours in sitting and SB raises concerns with regards to progression of potential cardio-metabolic diseases and co-morbidities in MD.

## Introduction

Muscular Dystrophy (MD) is a broad range of conditions, characterised by muscle wasting and progressive muscle weakness, and typically resulting in an eventual loss of ambulation [11]. Exercise was historically not recommended in these conditions for fear of exacerbating the condition progression [2], more recently however, adults with MD have been encouraged to exercise where possible, on the principle that “anything is better than nothing” [3]. Although there is increasing evidence of the benefits of exercise and physical activity in MD [4–7], investigations and understanding of sedentary behaviour (SB) in MD remains limited [8].

Sedentary behaviour is defined as energy expenditure of <1.5 metabolic equivalent of tasks (METs), and characterised by behaviours such as sitting, and watching TV [9]. Daily sitting time in particular has been a focus of SB research, and is associated with increased mortality risk, with Chau, Grunseit [10] reporting a 5% increased mortality risk, with every hour of sitting in excess of 7 hours a day, in the general population. Cardio-metabolic markers associated with cardiovascular and metabolic diseases are also widely recognised to increase with extended SB and specifically sitting time [11–15]. Sedentary behaviour and sitting time, and therefore their associated negative health consequences may be predisposed and exacerbated in MD, given the recognised muscle weakness and fatigue associated with the condition [16].

Systematic reviews have reported up to 53 studies assessing physical activity in adults with MD, within these reviews however, no reports of SB have been identified [8,17]. Recently, SB has been shown to occupy 83–89% of waking hours in a mixed group of ambulatory and non-ambulatory adults with MD, compared to 59% SB time in healthy controls [18]. The inclusion of both ambulatory and non-ambulatory adults with MD may be problematic in the assessment of SB, as SB time is known to be 20% lower in non-ambulatory compared to ambulatory boys with Duchenne MD (SB time = 91% in Non-Ambulant, and 71% in Ambulant) [19]. In addition to SB determined through METs, advances in accelerometry have now allowed posture classification, time spent Sitting or Standing in waking hours, to be identified [20], which has only previously been reported (9.1 Hours) using self-report methods in ambulant adults with MD [21]. Self-report methods however have been demonstrated to underestimate negative health behaviours such as SB and sitting time by up to 40%, when compared to objective assessments using accelerometers [22].

Despite exercise training and physical activity being promoted in ambulatory adults with MD [3], the barriers remain considerable [23], to this end, greater insight into SB, and sitting time in ambulant adults with MD may provide a physical behaviour that could be the target of future interventions, and reduce associated negative health outcomes. Therefore, this study aims to 1) objectively assess sedentary behaviour and physical activity in ambulant adults with MD, and 2) Assess sitting and standing time in ambulant adults with MD in comparison to a healthy control group, through the use of accelerometry.

## Methods

This study involves 14 ambulant adult males with MD, consistent with other cross-sectional studies of PA in MD [24–26], and 12 healthy males (CTRL). Adults with MD condition can be sub-classified as Beckers (N = 7), Fascioscapulohumeral (N = 6) and Limb-Girdle (N = 1), however evidence suggests no differences in physical behaviour between MD classifications [18], therefore all were grouped into a single MD group. All MD participants were recruited from, and tested at, The Neuromuscular Centre (Winsford, UK), with CTRL participants recruited from Manchester Metropolitan University and the surrounding area. CTRL participants were self-described as recreationally active, while no MD or CTRL participants were reported to be undertaking any structured exercise training programme. All participants signed an informed consent form prior to participation, following ethical approval being obtained through the Department of Sport and Exercise Science local Ethics committee, Manchester Metropolitan University. All procedures complied with the World Medical Association Declaration of Helsinki [27].

### Protocol

All participants underwent 7-day physical behaviour assessment using accelerometry, following anthropometric assessment. The same equipment was used for all participants. Stature and Mass were assessed by stadiometer (Harpenden, Holtain, Crymych, UK) and digital scales (Seca model 873, Seca, Germany), respectively. Upon completion of anthropometric assessment, MD participants’ physical function was assessed using the Swinyard Severity Classification scale [28] and 10 m walk time. Participants then had a wrist-worn accelerometer on the self-reported dominant arm for seven consecutive days (GENEActiv, Cambridge, United Kingdom).

### Body composition

Body composition was assessed using Biolectrical Impedance Analysis (BIA) (BodySTAT, 1500). All participants were assessed in a supine position, having completed no vigorous exercise in the previous 12 hours and at least two-hours postprandial. Electrodes were placed on the dorsal surfaces of metacarpals and metatarsals, along with between the styloid processes of the right ulna and radius, and between the medial and lateral malleoli of the right ankle. Participant anthropometrics were entered into the monitor, and the participant was asked to remain still. Absolute (Kg) and Relative (%) measures of participant Body fat and Fat Free Mass were produced. BIA is a common, reliable and validated measure to assess body composition in MD [29,30], due to impracticalities associated with other methods of assessment in a population that experience muscle weakness and some mechanical difficulties.

### Accelerometer

Physical Behaviour monitoring was successfully completed by all participants over 7 days, with 0 non-wear time. The tri-axial accelerometers were worn on the wrist rather than hip or thigh in order to increase adherence, as demonstrated in MD and non-MD populations [19,31,32].

Monitors were initialised to collect data at 100Hz for 7 days, with acceleration values (g’s) recorded continuously on each axis (X, Y and Z). Once the 7 days from initialisation had been completed, the monitors automatically stopped recording data, participants were then notified that they could remove the accelerometer from the wrist, and return the monitor to the principal investigator at their earliest convenience (for MD participants this was their next visit to The Neuromuscular Centre). Data was downloaded into a.bin file upon the monitors return, and converted in to 15s Epoch.csv files (GENEActiv PC Software, Version 3.2, Cambridge, United Kingdom). 15s Epochs were then analysed using the Wrist-Worn Sedentary Sphere [20] open access spreadsheet (https://www.researchgate.net/project/AMBer-Assessment-of-Movement-Behaviours), which assesses posture classification (either Sitting or Standing), as well as Sleep Hours, and Waking hours Physical Activity (consisting of Sedentary, Light, Moderate and Vigorous Activity using cut-points established by Esliger at al. [33]). As these physical activity cut off points were established for healthy adults and have not been validated in MD, all physical activity was grouped (Light + Moderate + Vigorous). Data is presented as a 7-day average of absolute hours of posture classification (Sitting or Standing) and absolute hours of SB or Physical Activity, and relative (%) waking hours of posture classification (Sitting + Standing = 100%), and relative (%) waking hours of SB or Physical Activity (SB + Physical Activity = 100%).

### Function

Overall physical function rather than specific measures of strength measures were used due to the heterogeneity of location and severity of muscle weakness in a cross MD classification sample.

#### Functional status

A chartered Neuromuscular Physiotherapist assessed all MD participants function using the Swinyard Disability Scale [28]. The Swinyard Disability Scale grades individuals ability to carry out activities of daily living from Stage 8 “Unable to sit without considerable support, requires maximal assistance for activities of Daily Living”, to Stage 1 “Mild abnormalities in gait, able to climb stairs without assistance. The Swinyard Disability Scale was originally validated in progressive muscular and neuromuscular diseases, and has been used extensively to describe functional status in adults with Muscular Dystrophy [34–36].

#### 10 m walk time

A 10 m walk test was performed by MD participants only. The 10 m walk test was run by a chartered physiotherapist on an even, carpeted surface. All participants started in a standing position on the start line, and were instructed to walk as safely and quickly to the finish line from the point of “Go”. Time was recorded with a stopwatch from the point of “Go” to the participant crossing the finish line. Those participants whom used ambulatory aids were allowed to complete the 10 m walk with their aid.

### Statistical analysis

IBM Statistics Version 26 (IBM SPSS, Armonk, New York) was used to perform all analyses, with a critical level of significance of 5%. Tests for parametricity were carried out on all data, with the exception of Ambulatory Assistance, Functional Assessment, 10m walk and employment information which are used to describe the populations only. All data was deemed parametric, with the exception of body mass which was Non-Parametric. Independent T-Tests were used to compare MD and CTRL groups for all parametric variables, with Mann Whitney U used to compare Body Mass. Associations between absolute measures of physical behaviour and body composition were performed, used Pearson’s Correlation for parametric variables and Kendall tau correlation for non-parametric variables. Independent MD Classification is presented for descriptional purposes only, with no statistical analysis.

## Results

### Anthropometrics

No differences were identified between MD and CTRL groups for Age, Stature or Body Mass (Table 1. *p* > 0.05).

**Table 1:** Participant characteristics.

	MD	*FSHD*	*BMD*	*LGMD*	CTRL
**N**	14	*6*	*7*	*1*	12
**Age (Years)**	37.8 (12.1)	*42*.*2 (12*.*5)*	*33*.*7 (12*.*2)*	*40*	36.3 (14.1)
**Stature (cm)**	179.1 (7.9)	*181*.*0 (11*.*0)*	*177*.*3 (17*.*3)*	*180*	180.6 (7.8)
**Body Mass (Kg)**	86.5 (45–115)	*86*.*5 (68–87)*	*71*.*8 (45–100)*	*115*.*1*	85.7 (18.0)
**Ambulatory Assistance**	4/14	*1/7*	*2/7*	*1/1*	-
**Functional Assessment**	1 (1–3)	*1 (1–2)*	*1 (1–3)*	*3*	-
**10 m Walk (s)**	10.0 (4.4)	*- 10*.*0 (5*.*4)*	*- 9*.*6 (4*.*1)*	*-13*.*0*	-
**Employment Status**					
Student	0/14	*0/6*	*0/7*	*0/1*	2/12
Part Time	5/14	*3/6*	*2/7*	*0/1*	0/12
Full Time	9/14	*3/6*	*5/7*	*1/1*	10/12

Characteristics of MD and CTRL Participants. All data presented as Mean (SD) with the exception of Functional Assessment and Body Mass, which are presented as Median (Range). MD = Muscular Dystrophy, CTRL = Control, Cm = Centimetres, Kg = Kilograms.

### Physical behaviour and posture classification

Absolute Sleeping Time was greater (1.3 Hours, *p* = 0.041) and Absolute Waking time was lower (1.3 Hours, *p* = 0.039) in adults with MD compared to CTRL. Absolute Sedentary Behaviour (2.2 Hours, *p* = 0.025) was greater in adults with MD compared to CTRL and Absolute Physical Activity (3.4 Hours, *p* < 0.001) was lower in adults with MD compared to CTRL (See Table 2). Absolute Sitting Time (1.9 Hours, *p* = 0.030) was greater in adults with MD compared to CTRL, and Absolute Standing Time (3.2 Hours, *p* < 0.001) was lower in adults with MD compared to CTRL (See Table 2).

**Table 2:** Accelerometer determined physical behaviour and posture classification (absolute).

		MD		*FSHD*		*BMD*	*LGMD*	CTRL
					Physical Behaviour			
Sleep Time (Hours)		8.7 (1.6)*		*8*.*2 (1*.*5)*		*9*.*2 (1*.*7)*	*7*.*7*	7.4 (1.3)
Waking Time (Hours)		15.3 (1.6)*		*15*.*8 (1*.*5)*		*14*.*8 (1*.*7)*	*16*.*3*	16.6 (1.3)
Sedentary Behaviour (Hours)		11.6 (2.3)*		*12*.*2 (1*.*6)*		*10*.*7 (2*.*7)*	*13*.*6*	9.4 (2.2)
Physical Activity (Hours)		3.8 (1.0)***		*3*.*6 (0*.*7)*		*4*.*1 (1*.*2)*	*2*.*6*	7.2 (2.2)
					Posture Classification			
Sitting Time (Hours)		9.9 (2.1)*		*9*.*9 (1*.*4)*		*9*.*9 (2*.*8)*	*10*.*4*	8.0 (2.2)
Standing Time (Hours)		5.4 (1.3)***		*5*.*9 (0*.*8)*		*5*.*0 (1*.*6)*	*5*.*9*	8.6 (2.3)

Physical Behaviour and Posture Classification comparison between adults with MD and CTRL. MD = Muscular Dystrophy, CTRL = Controls

* = *p* < 0.05

** = *p* < 0.01

*** = *p* < 0.001.

**Fig 1:**
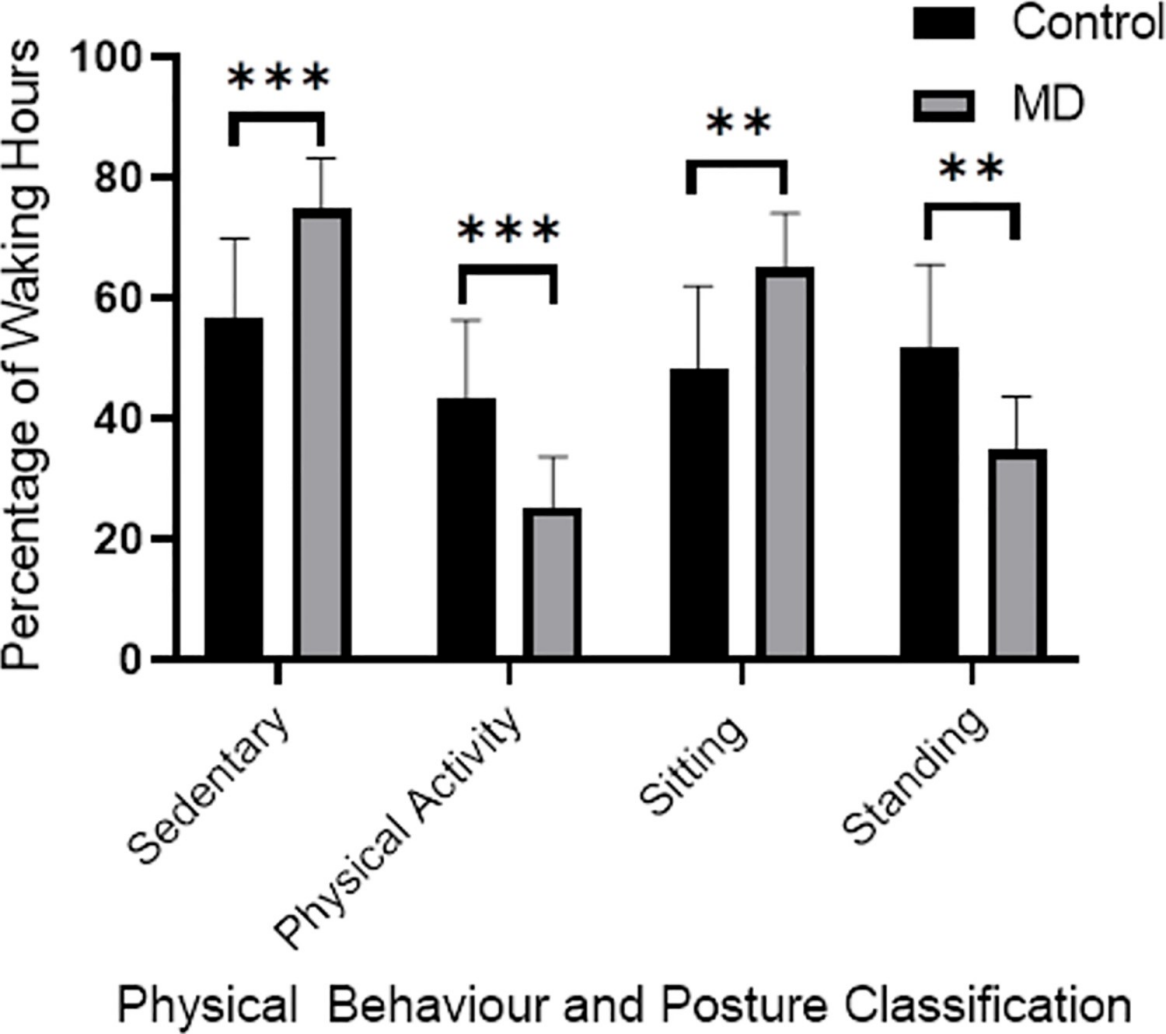
Daily physical behaviour and posture classification relative to waking hours (%). MD = Muscular Dystrophy, CTRL = Control, ** = *p* < 0.01, *** = *p* < 0.001.

Relative Sedentary Behaviour was 18 percentage points greater in adults with MD compared to CTRL (*p* < 0.001), and Physical Activity was 18 percentage points lower in adults with MD compared to CTRL (*p* < 0.001). Relative (percentage of Waking Hours) Sitting Time was 17 percentage points greater in adults with MD compared to CTRL (*p* = 0.001) and Relative Standing Time was 17 percentage points lower in adults with MD compared to CTRL (*p* = 0.001, See Fig 1).

### Associations between physical behaviour and posture classification with body composition and function

Absolute hours of Physical Activity in ambulatory adults with MD was associated with both Fat Mass (Kg) (R = -0.654, *p* < 0.05), Fat Mass (%) (R = -0.585, *p* < 0.05), 10m walk time (R = -0.546, *p* < 0.001) and Functional Status (R = -0.502, *p* < 0.05). Absolute hours of SB was associated with Fat Mass (Kg) (R = 0.643, *p* < 0.05), and absolute hours of standing was associated with 10m walk time (R = -0.547, *p* = < 0.05), in ambulatory adults with MD. No other associations were identified between measures of physical behaviour and body composition or function in ambulatory adults with MD (*p* > 0.05, See Table 3).

**Table 3:** Physical behaviour and posture classification associations with body composition and function.

	Physical Behaviour		Posture Classification	
	SB (Hours)	Physical activity (Hours)	Sitting (Hours)	Standing (Hours)
**Body Mass (Kg)**	0.258	-0.146	0.090	0.281
**BMI**	0.370	-0.344	0.082	0.239
**Fat Mass (Kg)**	**0.643** *	**-0.654** *	0.287	0.095
**Fat Mass (%)**	0.503	**-0.585** *	0.281	-0.083
**FFM (Kg)**	0.316	-0.115	0.065	0.368
**10m Walk (s)**	0.493	**-0.546** ***	0.486	**-0.547** *
**Functional Status**	0.177	**-0.502** *	0.030	-0.162

Physical Behaviour and Posture Classification Associations with Body Composition in ambulant adults with MD. MD = Muscular Dystrophy

* = *p* < 0.05

** = *p* < 0.01

*** = *p* < 0.001.

## Discussion

This study has objectively assessed physical behaviour through tri-axial accellerometry in a group of ambulant adults with Muscular Dystrophy, in comparison to CTRL. Tri-axial accelerometry has identified 118% more waking hours spent being sedentary and 18% less waking hours performing Physical Activity, in ambulant adults with MD. Uniquely, this assessment method has also used posture classification to identify 3.2 hours more (absolute) sitting time and 3.4 hours reduced (absolute) standing time in ambulant adults with MD compared to CTRL.

The CTRL group in the current study has presented comparable physical behaviours of ~8 hours of sitting to those reported by healthy adults in previous large cohort studies [37,38], while the SB (~9 hours) was slightly higher than the recommended 8-hours a day [39,40]. The ~11 hours of sedentary behaviour in ambulant adults with MD remained significantly higher than CTRLS, and may pose negative health implications beyond those directly associated with the MD conditions. Unsurprisingly, the inclusion of ambulant MD participants only in the present study has demonstrated a lower daily Sedentary Behaviour (75%) compared to those reported previously (82–85%) that included non-ambulant participants also [18]. To the authors knowledge this is the first work to objectively assess sitting time in ambulatory adults with MD, as such comparisons are difficult to make. The 9.9 hours reported here is greater than the 9.1 hour self-reported in adults with Limb-Girdle MD alone [21], however that study did not include any objective analysis of sitting time, nor information regarding the functional status of participants.

The present authors previous work has suggested Physical Activity interventions may be beneficial to attenuate functional decline in adults with MD [35]. Beyond lower physical activity, the present study highlights specifically that extended time in SB for ambulant adults with MD may have negative health consequences. In the present study, we observed associations between sedentary time and body fat, consistent with our previous work that denoted differences in fat mass between ambulant and non-ambulant adults with MD [41]. An accumulation of fat mass from sedentary behaviour has known health implications for the general population [42], such as hypertension, impaired glucose tolerance and fatigue, all characteristics associated MD [16,43]. Furthermore, the work from Chau et al. [10] would suggest a 15% increased mortality risk from the increased sitting time alone, a significant health burden. Ekelund et al. [44] noted that 60 minutes of moderate-vigorous physical activity would be required per day to offset the increased mortality risk from more than 8 hours of sitting per day in the general population, a feat unlikely in a progressive muscle condition such as MD. Within MD specifically, high levels of sedentary and sitting time may accelerate the development of ambulatory impairments as body fat accumulates alongside a deteriorating muscle mass, both factors we have previously observed to contribute to co-morbidities in adults with MD such as glucose intolerance, and lower QoL [16,43].

A wide range of research has evidenced benefits of reducing, interrupting and displacing SB in young and elderly populations [45–48]. Sitting, as a characteristic of SB, can be more identifiable behaviour than the generic concept of SB, which can make it an easier behaviour to break [49]. Future work should identify factors that contribute to increased sitting in ambulant adults with MD, with muscle weakness and experienced fatigue two likely factors [16], but also the implications of sitting and SB on cardio-metabolic health in adults with MD, and identification of behavioural interventions that may be effective to break and displace sitting/SB in adults with MD. The breaking of SB may represent a more accessible and safe means of positive health behaviours in ambulant adults with MD, given barrier to exercise may be considerable.

### Limitations

The authors acknowledge that participants recruited are regular attenders to a rehabilitation centre, for which clinical referral is required, as such this may predispose the sample to being more affected by MD, inducing greater SB. Alternatively, by virtue of regular attendance at a rehabilitation centre, this may suggest that this sample are more likely to want to engage in physical activity and condition management. In addition, this preliminary study has not identified the implications of increased sitting time and SB in ambulant adults with MD. Future studies should identify associations of physical behaviour with non-communicable health consequences such as cardio-metabolic biomarkers, as well as body composition identified in the present study. In addition, greater depth of understanding of physical behaviour in MD is required, such as the length of single bouts of sedentary behaviour, as well objective assessment of physical behaviours in other forms of MD, such as children with Duchenne.

In conclusion, this study has presented physical behaviour and posture classification in ambulant adults with MD in comparison to a healthy control group. Extended waking hours in sitting and SB has been demonstrated and raises concerns with regards to progression of potential cardio-metabolic diseases in MD. Future research needs to assess the implications of SB and sitting on health in adults with MD, and develop clear public health messaging to focus on SB as well as Physical Activity in neuromuscular disorders.

## Supporting information

S1 File (XLSX)
